# Effect of antioxidants on the H_2_O_2_-induced premature senescence of human fibroblasts

**DOI:** 10.18632/aging.102730

**Published:** 2020-01-21

**Authors:** Natalia Pieńkowska, Grzegorz Bartosz, Monika Pichla, Michalina Grzesik-Pietrasiewicz, Martyna Gruchala, Izabela Sadowska-Bartosz

**Affiliations:** 1 Department of Analytical Biochemistry, Institute of Food Technology and Nutrition, College of Natural Sciences, Rzeszow University, Rzeszow, Poland; 2 Department of Molecular Biophysics, Faculty of Biology and Environmental Protection, University of Lodz, Lodz, Poland; 3 Cytometry Lab, Department of Molecular Biophysics, Faculty of Biology and Environmental Protection University of Lodz, Lodz, Poland

**Keywords:** stress-induced premature senescence, fibroblasts, antioxidants, hydrogen peroxide

## Abstract

The study was aimed at evaluation of the role of secondary oxidative stress in the stress-induced premature senescence (SIPS) of human fibroblasts induced by H_2_O_2_. Two fibroblast lines were used: lung MRC-5 and ear H8F2p25LM fibroblasts. The lines differed considerably in sensitivity to H_2_O_2_ (IC_50_ of 528 and 33.5 μM, respectively). The cells were exposed to H_2_O_2_ concentrations corresponding to IC_50_ and after 24 h supplemented with a range of antioxidants. Most of antioxidants studied slightly augmented the survival of fibroblasts at single concentrations or in a narrow concentration range, but the results were not consistent among the cell lines. Chosen antioxidants (4-amino-TEMPO, curcumin, caffeic acid and *p*-coumaric acid) did not restore the level of glutathione decreased by H_2_O_2_. Hydrogen peroxide treatment did not induce secondary production of H_2_O_2_ and even decreased it, decreased mitochondrial potential in both cell lines and induced changes in the mitochondrial mass inconsistent between the lines. Antioxidant protected mitochondrial potential only in H8F2p25LM cells, but attenuated changes in mitochondrial mass. These results speak against the intermediacy of secondary oxidative stress in the SIPS induced by H_2_O_2_ and suggest that the small protective action of antioxidants is due to their effects on mitochondria.

## INTRODUCTION

Aging is an irreversible process affecting all higher organisms, characterized by progressive deterioration leading to a loss of function of cells, tissues, organs and finally death. Cellular aging can be accelerated by using non-lethal stresses; this phenomenon is referred to as stress induced premature senescence (SIPS). The concept of SIPS was first introduced in 2000 by Dr. Olivier Toussaint and co-workers [1, 2]. Premature aging of cultured cells is usually associated with the exposure of cells to environmental stress factors. Stress induced premature senescence can be defined as the long-term effect of sub-cytotoxic stress on proliferative cell types including appearance of many features of replicative senescence. Various genotoxic agents, such as hydrogen peroxide (H_2_O_2_), *tert*-butyl hydroperoxide, copper sulfate, diperoxovanadate, ethanol, mitomycin C, other cytostatic drugs, heat shock or UV radiation are well-established inducers of SIPS. It has been suggested that SIPS can be used in toxicology to identify xenobiotics that may induce premature senescence. In each case, oxidative stress is believed to be the major cause of SIPS program activation in normal cells [2–6].

Up to now, among other stressors, H_2_O_2_ is perhaps the best candidate for inducing senescence, because an H_2_O_2_-induced process might mimic the oxidative environment that may occur *in vivo* [2, 7, 8]. Addition of a single bolus of H_2_O_2_ to cultured cells means a rather short exposure to an external reactive oxygen species (ROS), which is rapidly decomposed [9, 10]. Hydrogen peroxide, which is plasma membrane permeable, may produce hydroxyl radical (^·^OH) in the presence of Fe^2+^ or Cu^2+^ through the Fenton reaction. Hydroxyl radical and the superoxide anion radical (O_2_^·-^) oxidize the unsaturated bonds of lipids to yield lipid peroxides as well as aldehydes such as 4-hydroxynonenal. Hydroxyl radical and lipid-derived aldehydes react with amino acid residues in proteins to produce carbonyl proteins [11] and modify nucleic acid bases [12]. Moreover, sublethal oxidative stress was shown to arrest proliferation and promote accumulation of senescence-associated molecular hallmarks [increased activity of cyclin-dependent kinase inhibitor p21Waf1/Cip1 (p21) and of acidic β-galactosidase (SA-β-gal), as well as diminution of phosphorylated retinoblastoma gene product (ppRb)] in human fibroblasts [13].

The causative role of oxidative stress in SIPS is well established [2–6, 14]. Nevertheless, it is of interest whether ROS play a role in secondary signaling leading to SIPS-induced cell death or if the execution of SIPS depends on the molecular machinery once triggered by oxidative stress and secondary production of ROS after initial oxidative stress is not important. One way to get an insight into this question is to examine the effect of antioxidants on human fibroblasts {on two human fibroblast lines (lung MRC-5 and H8F2p25LM fibroblasts, obtained from ear skin of an adult donor)} after H_2_O_2_ exposure and decomposition on the SIPS, which was the aim of this study. Our results speak for the second possibility.

## RESULTS

### Hydrogen peroxide sensitivity of fibroblasts

**Figure 1:**
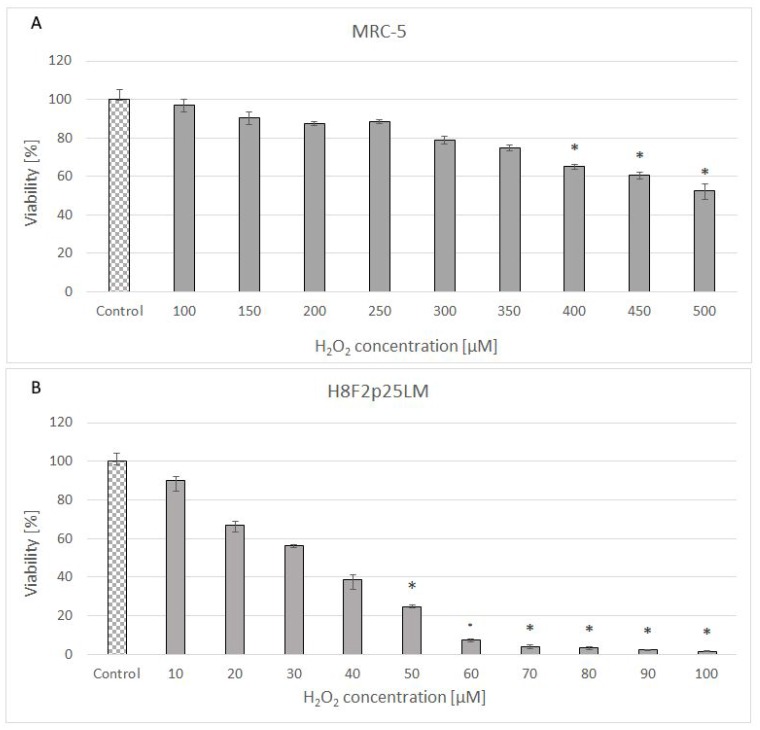
Viability of MRC-5 (**A**) and H8F2p25LM (**B**) cells after 24 h treatment with hydrogen peroxide at different concentrations estimated by Neutral Red assay. *P<0.05, Kruskal-Wallis test (against non-treated control).

**Figure 2:**
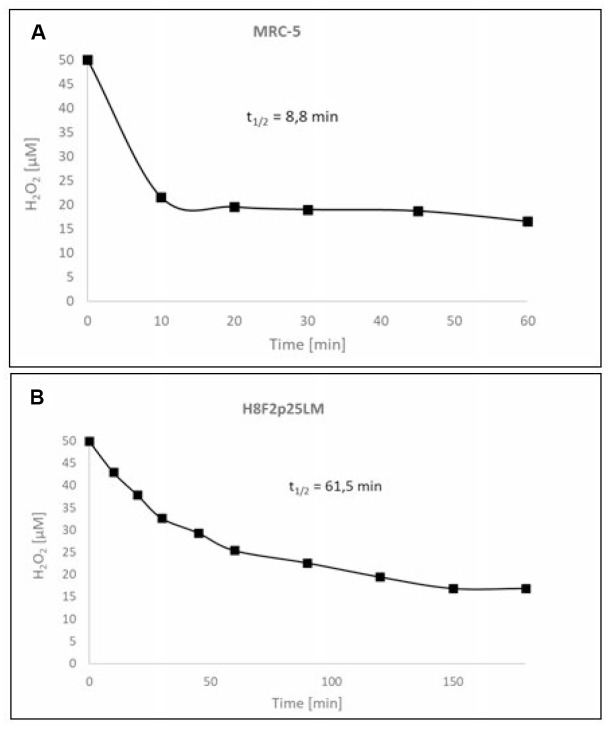
Kinetics of the decomposition of hydrogen peroxide by MRC-5 (**A**) and H8F2p25LM (**B**) cells.

Hydrogen peroxide showed a dose-dependent cytotoxicity against normal human fibroblast line [MRC-5 (CCL-171)] obtained from lung and primary human fibroblast line [H8F2p25LM] obtained from ear skin of an adult donor (Figure 1A, 1B). The cell lines differed considerably in the sensitivity to H_2_O_2_, with IC_50_ values of 528 and 33.5 μM for MRC-5 and H8F2p25LM fibroblasts, respectively, when estimated after 24 h. The more resistant MRC-5 cells more rapidly decomposed H_2_O_2_ than H8F2p25LM fibroblasts, the half-life times of 50 μM H_2_O_2_ in the presence of 5 x 10^3^ cells being 8.8 minutes for MRC-5 cells and 61.5 minutes for H8F2p25LM cells (Figure 2A, 2B). This difference was mainly due to different catalase activity, which was about 11 times higher in MRC-5 cells than in H8F2p25LM cells (28.03 and 2.56 μmol H_2_O_2_/(s*10^6^ cells), respectively).

### Protection of fibroblasts against the H_2_O_2_-induced cytotoxicity

**Figure 3:**
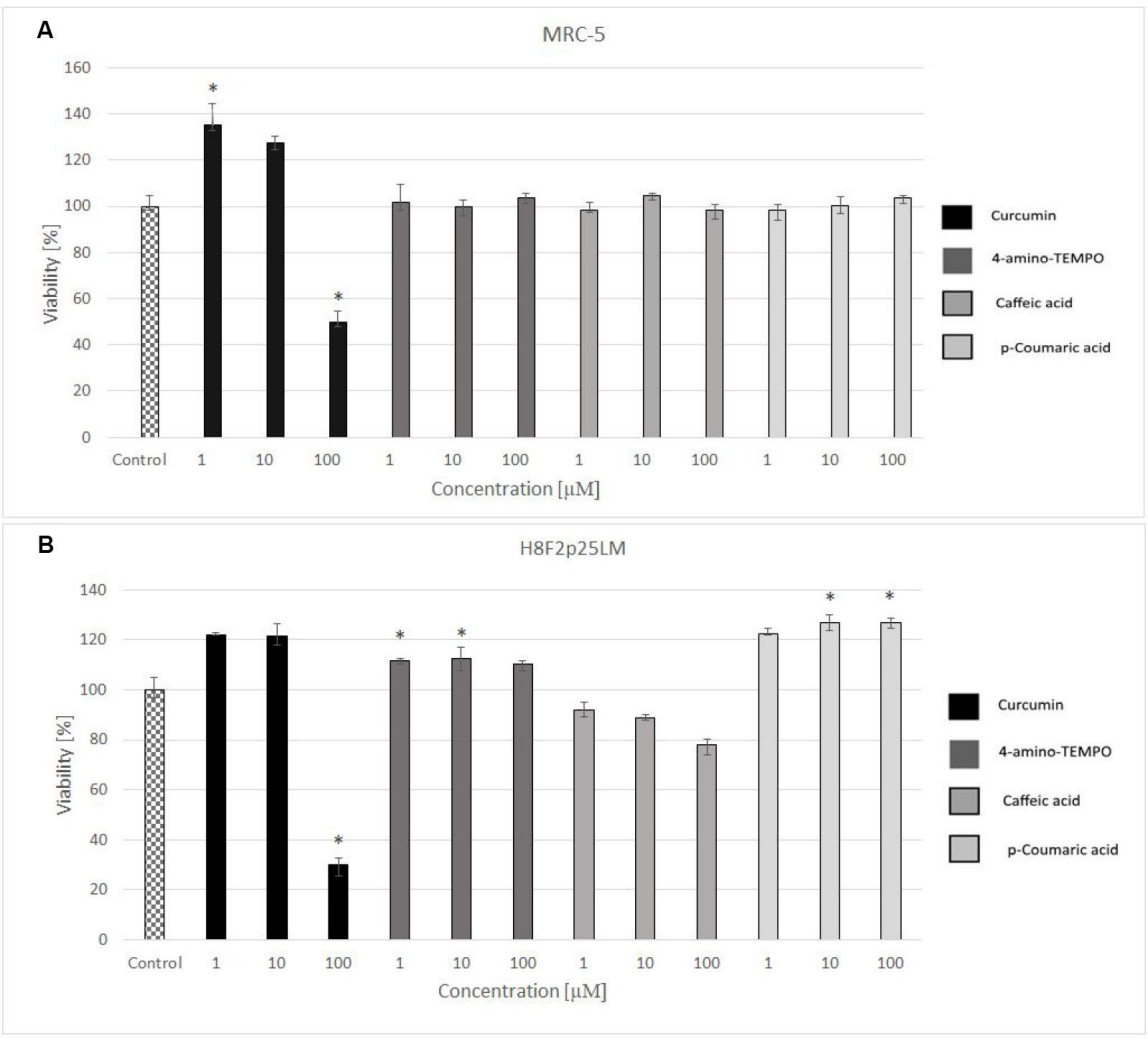
Viability of MRC-5 (**A**) and H8F2p25LM (**B**) cells after 24 h treatment with selected antioxidants (1, 10 and 100 μM), estimated by Neutral Red assay. *P<0.05, Kruskal-Wallis test (against non-treated control).

**Figure 4:**
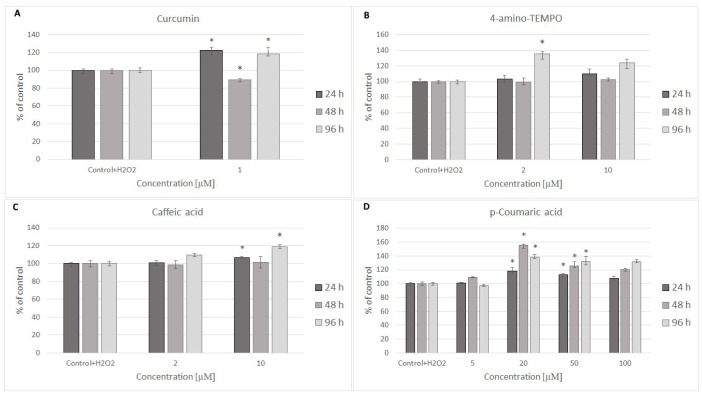
MRC-5 cell survival after 24 h treatment with 600 μM hydrogen peroxide and then 24 h, 48 h and 96 h posttreatment with different concentrations of curcumin (**A**), 4-amino-TEMPO (**B**), caffeic acid (**C**) and p-coumaric acid (**D**). *P<0.05, Kruskal-Wallis test (against control containing H_2_O_2_).

**Figure 5:**
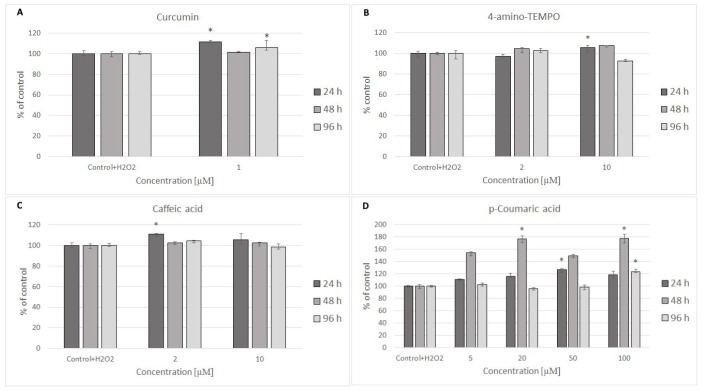
H8F2p25LM cell survival after 24 h treatment with 35 μM hydrogen peroxide and then 24 h, 48 h, 96 h posttreatment with different concentrations of curcumin (**A**), 4-amino-TEMPO (**B**), caffeic acid (**C**) and p-coumaric acid (**D**). *P<0.05, Kruskal-Wallis test (against control containing H_2_O_2_).

24 hours after H_2_O_2_ treatment, antioxidants were added to the cells to study their effects on the processes dependent on secondary oxidant-dependent signaling leading to decrease in cell survival. The antioxidants were first checked for their cytotoxicity (data for 4 chosen antioxidants are shown in Figure 3A and 3B) and used at non-toxic concentrations. Among the antioxidants tested, 2 μM 4-amino-TEMPO, 10 μM TEMPOL, 2-10 μM gallic acid, 10 μM caffeic acid, 50-100 μM aminoguanidine hydrochloride, 1 μM curcumin,5-100 μM oleuropein, 100 μM melatonin as well as 20-50 μM *p*-coumaric acid augmented the survival of MRC-5 cells, while 50-100 μM TEMPO, 10 μM 4-amino-TEMPO, 2 μM caffeic acid, 1 μM curcumin, 5 μM ethoxyquin, 20-100 μM *p*-coumaric acid and 50 μM ferulic acid increased the survival of H8F2p25LM cells after treatment with hydrogen peroxide (Figures 4A–4D and 5A–5D). On this basis, 4 compounds showing some protection of both cell lines were chosen for further experiments (data for only these compounds are shown in Figures 4A–4D and 5A–5D).

### Hydrogen peroxide generation by the antioxidants studied

We compared generation of hydrogen peroxide by the antioxidant compounds used in this study during incubation in the culture medium. Some of these compounds generated significant amounts of hydrogen peroxide (gallic acid > caffeic acid > oleuropein), which represents a pro-oxidant effect of these antioxidants. No detectable H_2_O_2_ generation was found for 4-hydroxy-TEMPO, 4-amino-TEMPO, TEMPO, curcumin, resveratrol, ethoxyquin, melatonin, ferulic acid, *p*-coumaric acid and aminoguanidine hydrochloride (Table 1). Both H_2_O_2_-generating compounds (caffeic acid, oleuropein) and compounds which did not produce H_2_O_2_ in the culture media offered small post-exposure protection against the effects of H_2_O_2_.

**Table 1:** Generation of hydrogen peroxide in DMEM/ DMEM + glutaMAX medium [(mean ± SD; n≥3 (independent samples)].

**Compound**	**H_z_O_2_ [μM]**	
	**DMEM**	**DMEM + glutaMAX**
Gallic acid	77.95±1.47	124.66 ± 5.41***
Caffeic acid	68.04±3.25	96.10 ± 3.71***
Oleuropein	64.87±1.63	81.42 ± 7.22*
Aminoguanidine chloride	0	4.52 ± 1.75*

The following substances did not produce detectable amounts of H_2_O_2_: *curcumin, resveratrol, ethoxyquin, melatonin, ferulic acid, p-coumaric acid, TEMPO,* *4-hydroxy-TEMPO and 4-amino-TEMPO.* Statistical significance of differences between generation of hydrogen peroxide in DMEM and DMEM + glutaMAX medium: *P < 0.05, **P < 0.01, ***P < 0.001.

### Glutathione content

**Figure 6:**
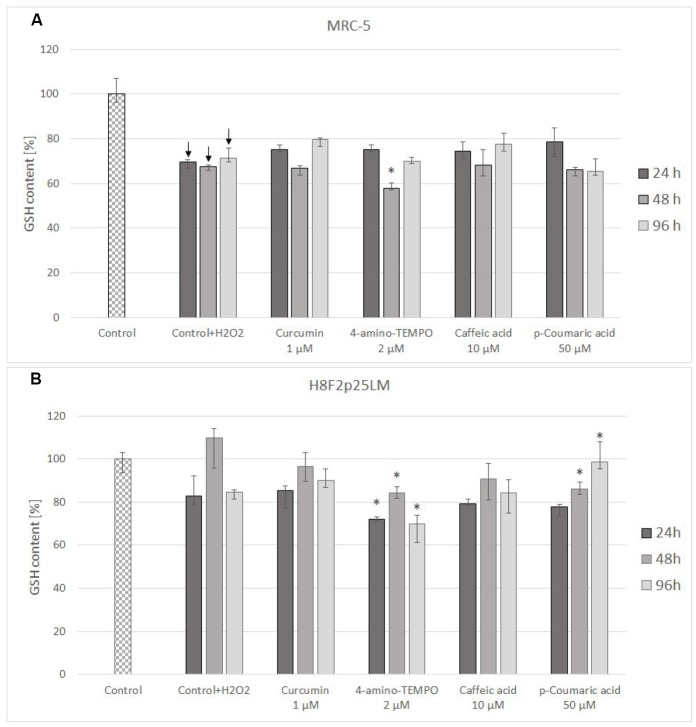
GSH content in MRC-5 (**A**) and H8F2p25LM (**B**) cells after 24 h treatment with hydrogen peroxide and 24 h, 48 h, 96 h posttreatment with selected concentrations of antioxidants. *P<0.05, Kruskal-Wallis test (against control treated only with H_2_O_2_) ↓ differences between treated and non-treated control.

Treatment with hydrogen peroxide decreased the content of reduced glutathione (GSH) in MRC-5 fibroblasts and did not cause a statistically significant decrease of GSH level in H8F2p25LM cells (although a tendency for decrease was visible; Figure 6A, 6B). Posttreatment exposure to the chosen antioxidants did not augment the GSH level with respect to cells treated with H_2_O_2_ only, while 4-amino-TEMPO and 50 μM *p*-coumaric acid evoked a further GSH depletion in H8F2p25 LM cells (Figure 6B).

### Reactive oxygen species

**Figure 7:**
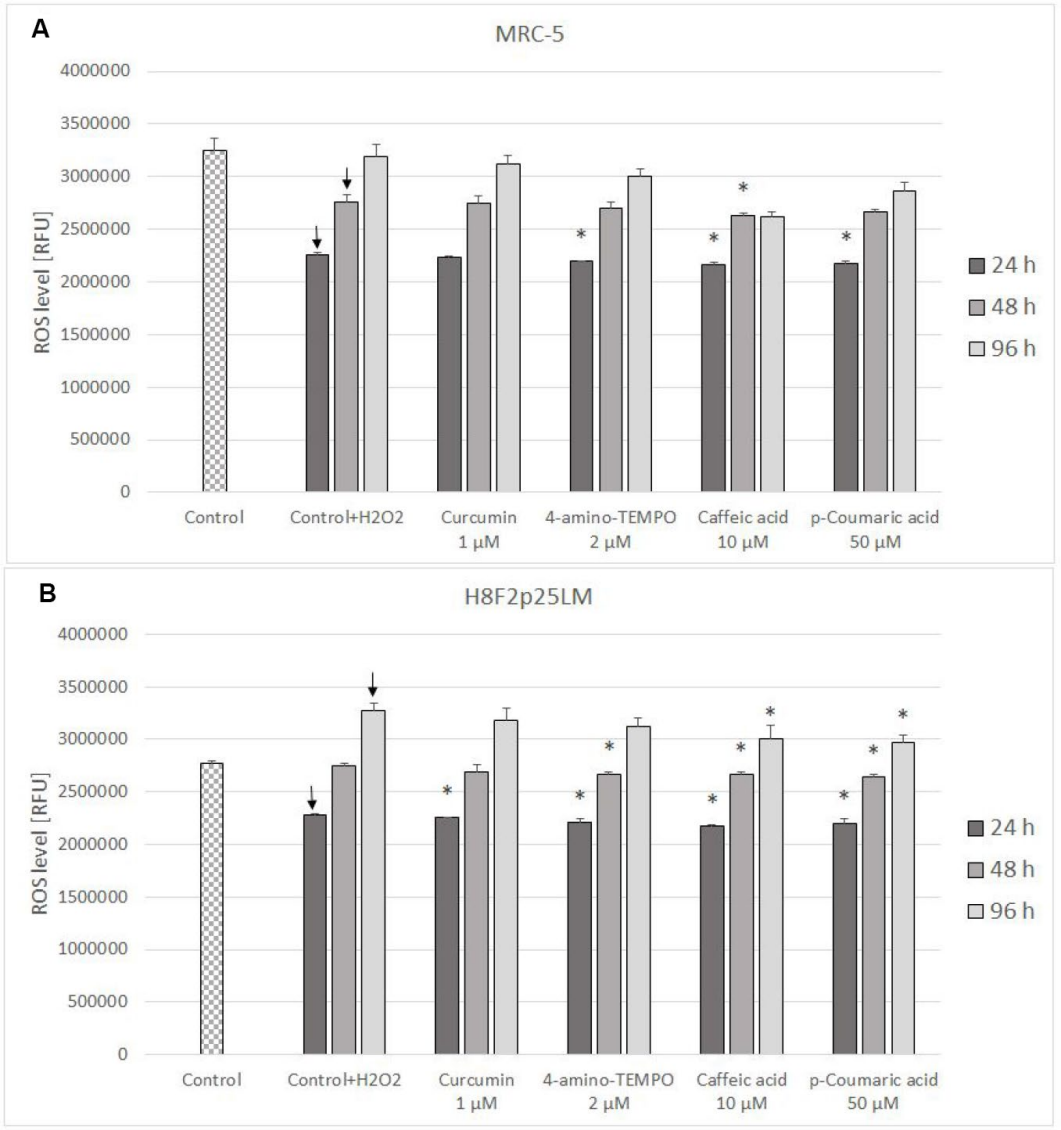
ROS level (estimated with H_2_DCFDA) in MRC-5 (**A**) and H8F2p25LM (**B**) cells after 24 h treatment with hydrogen peroxide and 24 h, 48 h, 96 h posttreatment with selected concentrations of antioxidants. * P≤0.05, t-Student test against H_2_O_2_ treated control; ↓ differences between treated and non-treated control.

The level of reactive oxygen species (ROS) estimated with H_2_DCF-DA decreased in H_2_O_2_-treated cells after next 24 h following the day of exposure and then increased gradually, exceeding the control level in H8F2p25LM cells. Posttreatment with antioxidants induced a small decrease in the ROS level in the majority of cases (Figure 7A, 7B).

**Figure 8:**
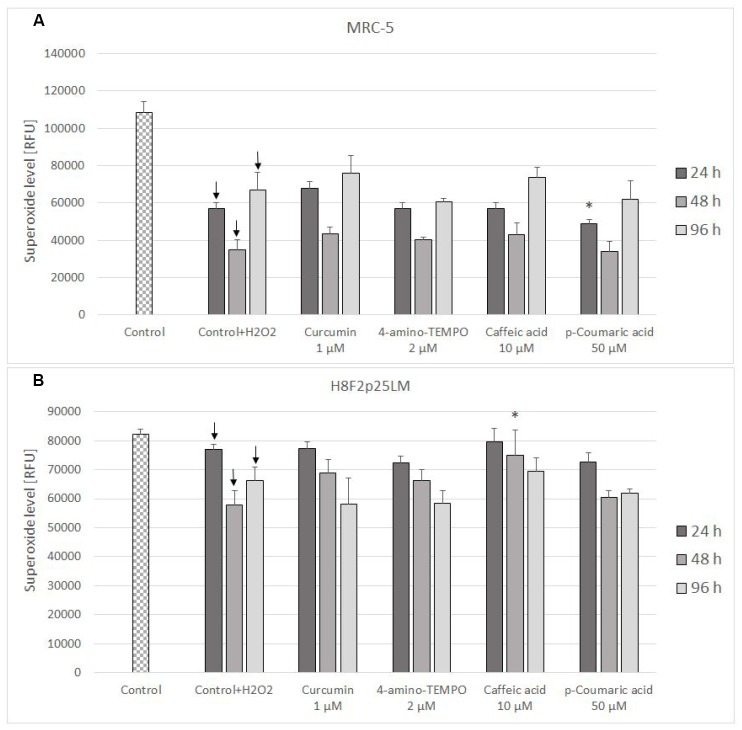
Mitochondrial superoxide level in MRC-5 (**A**) and H8F2p25LM (**B**) cells after 24 h treatment with hydrogen peroxide and 24 h, 48 h, 96 h posttreatment with selected concentrations of antioxidants. * P≤0.05 t-Student test against H_2_O_2_ treated control; ↓ differences between treated and non-treated control.

The level of mitochondrial superoxide decreased in H_2_O_2_-treated cells and was not significantly affected by antioxidant posttreatment (Figure 8A, 8B).

### Mitochondrial membrane potential and mitochondrial mass

**Figure 9:**
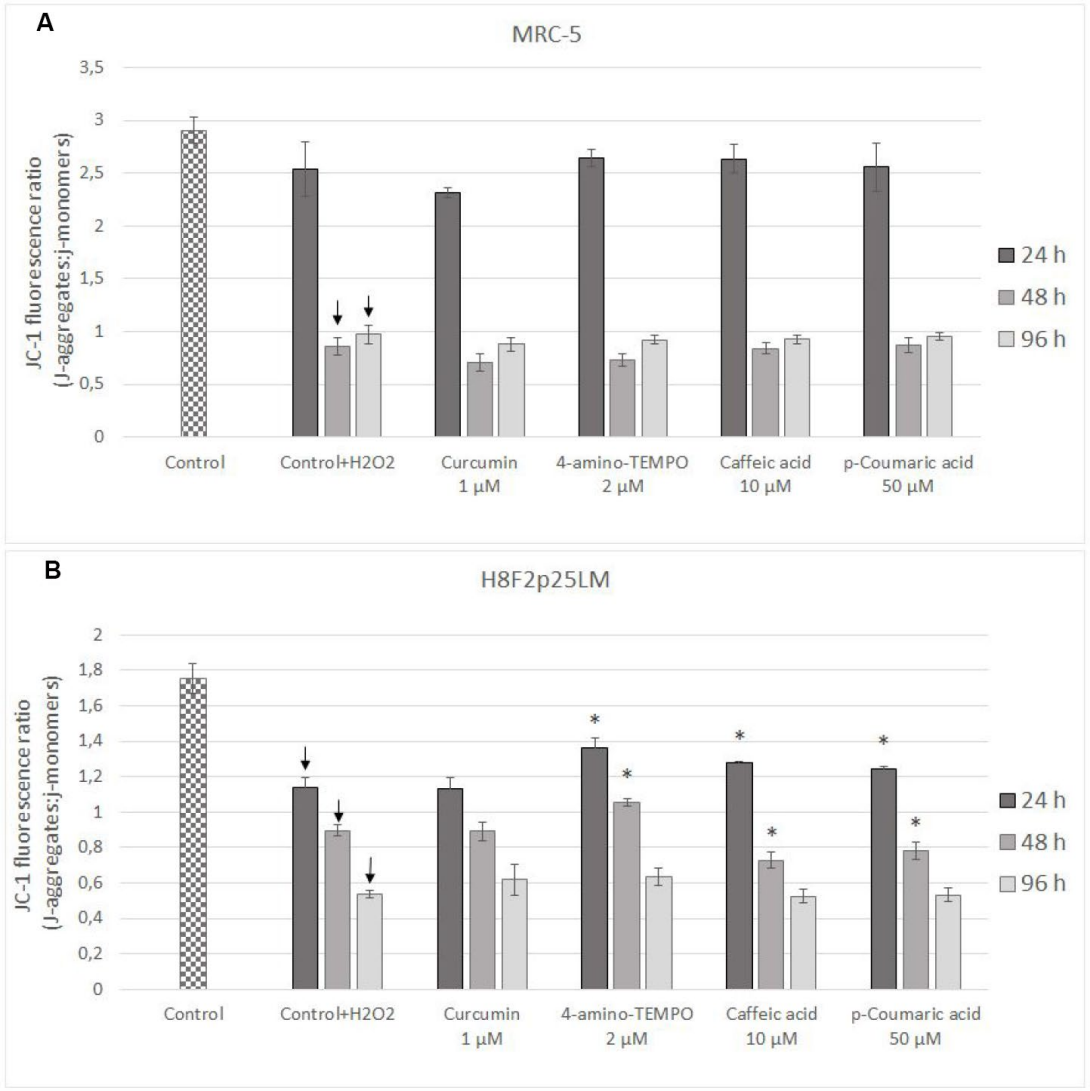
Changes in MRC-5 (**A**) and H8F2p25LM (**B**) cell mitochondrial potential after 24 h treatment with hydrogen peroxide and 24, h 48 h, 96 h posttreatment with selected concentrations of antioxidants. * P≤0.05 t-Student test against H_2_O_2_ treated control. ↓ differences between treated and non-treated control.

Estimation of changes in mitochondrial membrane potential (*Δψ_m_*) by JC-1 staining of cells demonstrated that the mitochondrial membrane potential was significantly reduced in H_2_O_2_ treated cells [in subsequent times after pretreatment: 24 h, 48 h and 96 h in H8F2p25LM cells and after 48 h as well as 96 h in MRC-5 cells; Figure 9A, 9B]. Only 4-amino-TEMPO after 24 h as well as 48 h, caffeic acid and p-coumaric acid after 24 h, respectively, showed some preventive effect in H8F2p25LM cell line. No effects of antioxidants on *Δψ_m_* were seen in MRC-5 cells.

**Figure 10:**
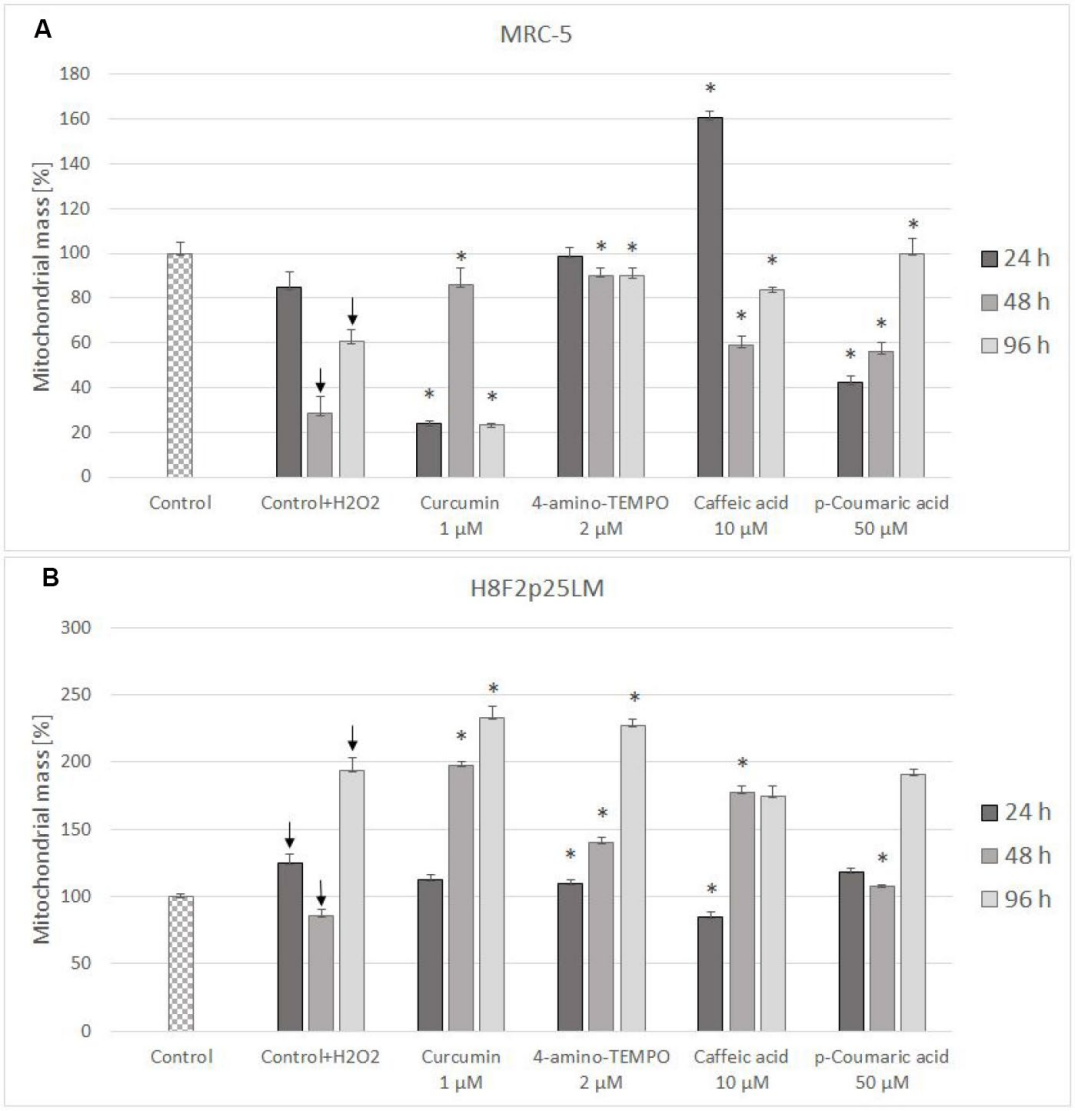
Changes in MRC-5 (**A**) and H8F2p25LM (**B**) cell mitochondrial mass after 24 h treatment with hydrogen peroxide and 24 h, 48 h, 96 h posttreatment with selected concentrations of antioxidants. * P≤0.05, t-Student test against H_2_O_2_ treated control; ↓ differences between treated and non-treated control.

Mitochondrial mass decreased in H_2_O_2_-treated MRC-5 cells (Figure 10A), but increased (except for the time of 48 h) in H8F2p25LM cells following H_2_O_2_ treatment (Figure 10B). Antioxidants were generally protective against these changes, except for curcumin after 24 h and 96 h and *p*-coumaric acid after 24 h in MRC-5 cells (Figure 10A), and 4-amino-TEMPO as well as caffeic acid after 24 h in H8F2p25LM cells(Figure 10B).

### Senescence-associated β-galactosidase

**Figure 11:**
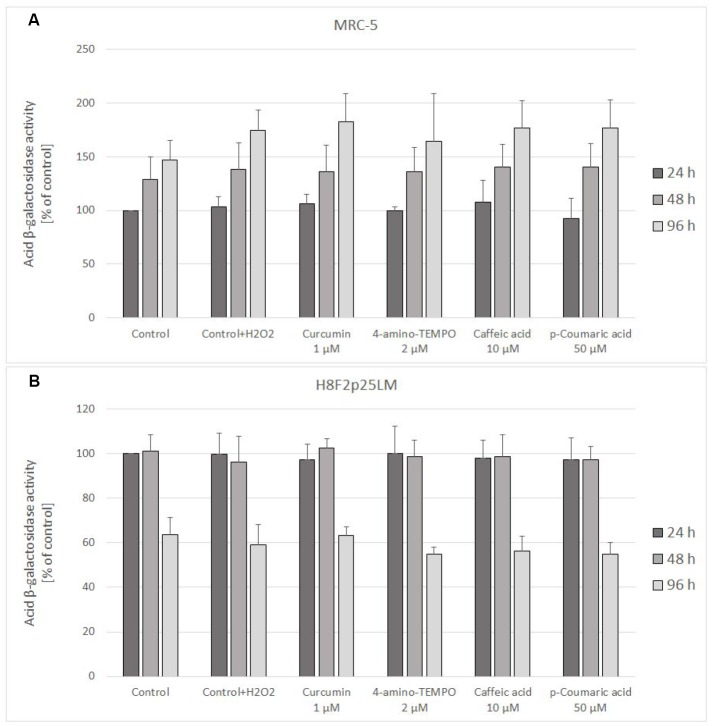
Senescence-associated β-galactosidase activity in MRC-5 (**A**) and H8F2p25LM (**B**) cells treated with hydrogen peroxide and posttreated with investigated compounds. Fluorescence value of control samples at 24-hour time point was considered 100% for each series. Data are presented as mean ± SD of 6 (MRC-5) or 2 (H8F2p25LM) independent experiments.

Staining for acidic β-galactosidase activity showed a gradual increase in the staining of H_2_O_2_-treated MRC-5 cells, which was not significantly affected by treatment with antioxidants. In H8F2p25LM cells, the staining was not increasing after 48 h and even decreased after 96 h. Again, no significant effects of the antioxidants was evident (Figure 11A, 11B).

## DISCUSSION

Dermal fibroblasts are long lived cells undergoing age-associated damage accumulation underlying the age-related changes in the skin. Skin wrinkles and sagging are important factors defining skin aging. Fibroblasts play a main role in the production of extracellular matrix components in the skin. Skin aging is the consequence of reduced numbers of fibroblasts, decreased skin elasticity and tonus as well as lower levels of extracellular matrix proteins, thus resulting in the formation of wrinkles [11, 15]. Fibroblasts play also a role in the accelerated aging of the skin induced by UV radiation and other environmental factors [16–17].

Fibroblasts are also model cells in studies of *in vitro* cellular aging and SIPS is an acknowledged model of accelerated aging *in vitro*, mimicking some aspects of accelerated skin aging *in vivo*. Our results indicate that there may be significant differences between different fibroblast lines in the sensitivity to H_2_O_2_ (Figure 1A, 1B) linked to the capacity to decompose this compound (Figure 2A, 2B).

Hydrogen peroxide is naturally produced in the human cells during many physiologic and pathological processes and has been widely used as a model pro-oxidant in the study of oxidative stress. It has been reported that ROS, especially H_2_O_2_ and superoxide anions, are associated with the cellular proliferation of many cell types including fibroblasts [18]. However, elevated levels of H_2_O_2_ induce premature senescence and H_2_O_2_ treatment is a standard procedure used for this purpose [2, 7, 8].

Both cell lines studied by us were treated with H_2_O_2_ at concentrations corresponding to their IC_50_ values. Oxidative stress has been repeatedly proposed to be the factor responsible for the induction of fibroblast SIPS and antioxidants have been reported to be protective. For example, pretreatment with rapamycin decreased the extent of SIPS induced by UV exposure and this effect was ascribed to the modification of antioxidant defense by rapamycin and suppression of free radical production by irradiation [19]. We were interested in checking whether the mechanism of SIPS-induced cell death is dependent on the secondary production of ROS and if antioxidants can modulate the course of cellular changes following exposure to H_2_O_2_. Literature data do not allow for reaching an unanimous answer to this question.

Gamma-tocotrienol, biodynes, tocotrienol-rich fraction (TRF) and tocopherol were found to be protective against changes in collagen synthesis and degradation caused by H_2_O_2_-induced SIPS, nonetheless the cells were pretreated with these compounds prior to exposure to H_2_O_2_ [20, 21]. *Centella asiatica* herb extracts containing various pentacyclic triterpenes protected fibroblasts against the effects of H_2_O_2_-induced SIPS but, again, the cells were pre-exposed to the extracts before exposure to H_2_O_2_ [22]. Malvidin [23], cyanidin [24] and phloroglucinol [25] were reported to protect fibroblasts from H_2_O_2_-induced SIPS and decrease the level of lipid peroxidation enhanced by H_2_O_2_ exposure. Nevertheless, in these experiments the compounds were introduced soon after exposure to H_2_O_2_, so the results do not allow for distinguishing between effects of primary and secondary oxidative stress, if the latter was involved in the signaling mediating the development of SIPS.

The SIPS induced by exposure to H_2_O_2_ is known to proceed in a different way and to involve different pathways than replicative senescence. In our study, the senescence-associated β-galactosidase activity, one of indices of replicative aging, observed in H_2_O_2_-treated cells, was increased in MRC-5 cells, but its increase was not modified by antioxidants (Figure 11A). No increase in the acidic β-galactosidase activity was seen in H8F2p25LM cells, which are more sensitive to H_2_O_2_ and were treated with a lower concentration of H_2_O_2_ (Figure 11B). Interestingly, no increased staining for the senescence-associated β-galactosidase was also reported for Werner syndrome fibroblasts, which are more sensitive to H_2_O_2_ than normal fibroblasts [26]. Perhaps, higher H_2_O_2_ concentrations are required for induction of increase in acid β-galactosidase activity than for triggering the main signaling pathways inducing the SIPS program. Results from other studies indicate that these pathways include NF-κB, iNOS, p53, COX-2, the caspase-3/keratin-18 pathway and serine/threonine kinase Aurora A/MDM2 pathway as well as proteasome/ubiquitin ligase pathways of protein degradation [23, 27]. Hydrogen peroxide-induced SIPS was also found to involve pancreatic ER kinase (PERK)-mediated upregulation of CCAAT/enhancer-binding protein homologous protein and activation of unfolded protein response [28].

Posttreatment with antioxidants provided small and inconsistent protection against the toxicity of H_2_O_2_ (Figures 4A–4D and 5A–5D). Antioxidants did not affect considerably changes in GSH level induced by H_2_O_2_ treatment (Figure 6A, 6B).

The treatment with H_2_O_2_ did not induce secondary production of ROS but, at least initially, rather decreased it. The effect of antioxidants on the ROS level in H_2_O_2_-treated cells was slight or none (Figures 7A, 7B and 8A, 8B).

Hydrogen peroxide treatment decreased mitochondrial membrane potential in both cell lines studied; antioxidants provided some protection only in H8F2p25LM cells, but not in MRC-5 cells (Figure 9A, 9B).

Changes in the mitochondrial mass were inconsistent between the lines, a decrease being observed in MRC-5 cells and mostly an increase in H8F2p25LM cells. In both lines antioxidants were generally protective with respect to these changes (Figure 10A, 10B).

In summary, these results speak against the intermediacy of secondary ROS formation in the SIPS induced by H_2_O_2_. In line with our results, Le Boulch et al. analyzed protein carbonylation in human fibroblasts in the course of SIPS induced by H_2_O_2_ exposure identifying an “Oxi-proteome”, i.e. a set of proteins that are building up as oxidized. However, they did not observe an increase in the overall level of protein oxidation during SIPS; rather, this level was decreasing during several days after exposure [29]. These results speak against increase in ROS production after H_2_O_2_ exposure, which should lead to an increase in the overall protein carbonylation.

The small protective action of antioxidants observed in our study is most probably due to their effects on mitochondria of H_2_O_2_-treated cells. The critical role of mitochondrial changes involving release of cytochrome c in the development of SIPS has been documented by others [20].

## MATERIALS AND METHODS

### Materials and equipment used in studies of cell-free systems

Xylenol Orange (cat. no. chem*237045902*5g) was obtained from Polish Chemical Reagents (POCh, Gliwice, Poland), perchloric acid (HClO_4;_ cat. no. chem*115649402*1l) was purchased from Chempur (Piekary Śląskie, Poland), phosphate-buffered saline (PBS: 145 mM NaCl, 1.9 mM NaH_2_PO_4_, 8.1 mM Na_2_HPO_4_, cat. no. PBS405) was obtained from Lab Empire (Rzeszów, Poland). Dulbecco’s Modified Eagle Medium + GlutaMax (DMEM+GlutaMax) (cat. no. 21885-025) and Dulbecco’s Modified Eagle Medium (DMEM) (cat. no. 12430-054) and Dulbecco’s Phosphate Buffered Saline (DPBS) (cat. no. 14040-117) were purchased from Thermofisher Scientific (Waltham, MA, USA). All other reagents, if not mentioned otherwise, were purchased from Sigma-Aldrich Corp. (St. Louis, MO, USA) and were of analytical grade. Distilled water was purified using a Milli-Q system (Millipore, Bedford, MA, USA). In cell free system analysis absorptiometric measurements were done in a Spark multimode microplate reader (Tecan Group Ltd., Männedorf, Switzerland). Distilled water was purified using a Milli-Q system (Millipore, Bedford, MA, USA).

### Assay of hydrogen peroxide generation

Protocol for evaluation of H_2_O_2_ generation by antioxidants consisted in addition of 18 μl of 10 mM antioxidant to 162 μl of DMEM/DMEM + glutaMAX + serum. The samples were incubated for 3 h at 37±1°C with shaking and the peroxide content was estimated before and after incubation by the ferric-Xylenol Orange method [30]. Then, to 180 μl samples, 20 μl of Xylenol Orange reagent was added [2.5 mM Xylenol Orange/2.5 mM Mohr’s salt [Fe_2_(NH_4_)_2_SO_4_; purity of 99.997 %] in 1.1 M perchloric acid]. After 30-min incubation at room temperature, absorbance of the samples was measured at 560 nm and hydrogen peroxide concentration was read from a standard curve.

### Materials and equipment used to study the fibroblast cell lines

Human lung normal fibroblast cell line [MRC-5 (CCL-171)] was obtained from American Type Culture Collection (ATCC). The MRC-5 cell line was derived from normal lung tissue of a 14-week-old male foetus. This is a normal diploid human cell line with 46, XY karyotype. Before the onset of senescence MRC-5 are capable of 42 to 46 population doublings; they were used between 20^th^ and 25^th^ population doublings. Primary human fibroblast [H8F2p25LM] were isolated from ear skin of an adult donor; they were used between the 3^rd^ and 7^th^ population doubling.

Dulbecco’s Modified Eagle Medium + GlutaMax (DMEM+GlutaMax) (cat. no. 21885-025), Dulbecco’s Modified Eagle Medium (DMEM) (cat. no. 12430-054) and Dulbecco’s Phosphate Buffered Saline (DPBS) (cat. no. 14040-117) were purchased from Thermofisher Scientific (Waltham, MA, USA). Phosphate-Buffered Saline (PBS) without Ca^2+^ and Mg^2+^ (cat. no. 02-023-1A), Trypsin-EDTA solution (10x) (cat. no. 03-051-5B), Foetal Bovine Serum (cat. no. 04-001-1A) and Penicillin-Streptomycin solution (cat. no. 03-031-1B) were obtained from Biological Industries (Cromwell, CT, USA). 0.33% Neutral Red (NR) solution (cat. no. N2889), 0.4% Trypan Blue solution (cat. no. T8154), *p*-coumaric acid (cat. no. C9008), caffeic acid (cat. no. C0625), TEMPO (cat. no. T-7263), 4-hydroxy TEMPO (cat. no. H8258), 4-amino TEMPO (cat. no. 163945), ferulic acid (cat. no. 128708), aminoguanidine hydrochloride (cat. no. 396494), oleuropein (cat. no. O8889), resveratrol (cat. no. R5010), gallic acid (cat. no G7384), *N*-ethylmaleimide (NEM) (cat. no. E3876), trichloroacetic acid (TCA) (cat. no. T4885), diethylenetriaminepentaacetic acid (DTPA) (cat. no. D1133), L-ascorbic acid (cat. no. A0278), 2′,7′-dichlorodihydrofluorescein (H_2_DCF-DA) (cat. no. D6883), dimethyl sulfoxide (DMSO) (cat. no. D2438), *ortho*-phtal-dialdehyde (OPA) (cat. no. P1378) were provided by Sigma-Aldrich (St Louis, MO, USA). 96 % ethanol (cat. no. 396420113), glacial acetic acid (cat. no. 568760114) as well as methanol (cat. no. 6219900110) were obtained from Avantor Performance Materials Poland. Hydrogen peroxide, 30% was purchased from CHEMPUR (Poland). Curcumin (cat. no. SC200509) was obtained from Santa Cruz Biotechnology. Melatonin (cat. no. MEL550.500) was purchased from Bioshop. Ethoxyquin (cat. no. 02810) was provided by Fluka.

Cell culture: 75 cm^2^flasks (cat. no. 156499) were provided by Thermofisher Scientific (Waltham, MA, USA). Transparent 96-well culture plates (cat. no 655180) and black flat bottom 96-well plates (cat. no. 655209) were obtained from Greiner (Kremsmünster, Austria). Other sterile cell culture materials were provided by Nerbe (Winsen, Germany).

Stock solutions of antioxidants were freshly prepared in PBS (4-amino TEMPO, 4-hydroxy TEMPO, gallic acid, caffeic acid) or dimethyl sulfoxide (DMSO) (other antioxidants and filtered through a 0.22 μm filter before each experiment. The final highest concentration of DMSO in cell media was ≤ 0.02%, and had no significant effect on the treated cell lines. Absorptiometric and fluorometric measurements were done in a Spark multimode microplate reader (Tecan Group LTD., Männedorf, Switzerland). Measurements were performed in sixplicate.

### Cell culture

MRC-5 cell were cultured in DMEM + GlutaMax and H8F2p 25LM were cultured in DMEM supplemented with 1% v/v penicillin and streptomycin solution and 10 % heat inactivated foetal bovine serum (FBS). Cells were incubated at 37°C under 5% carbon dioxide and 95°C humidity. Cells were passaged at about 85% confluence. Cell morphology was examined under an Zeiss Primo Vert (Oberkochen, Germany) inverted microscope with phase contrast. Fibroblasts viability was estimated by Trypan Blue exclusion test. Cells were counted in a Thoma hemocytometer (Superior Mrienfeld, Lauda-Königshofen, Germany).

### The kinetics of hydrogen peroxide decomposition by fibroblasts

The fibroblasts were plated in wells of a multi-well plate. After 24 h the medium was removed and a new one containing H_2_O_2_ at a concentration of 50 μM was added. After 0, 10, 30, 45, 60, 120 and 180 minutes 90 μl of medium was collected from each well and the concentration of remaining H_2_O_2_ was determined.

To determine hydrogen peroxide, 10 μl of Xylenol Orange reagent was added to the collected 90 μl aliquots. After 30-min incubation at room temperature, absorbance of the samples was measured at 560 nm.

### Determination of hydrogen peroxide inhibitory concentration (IC_50_)

The cell were seeded in 96-well clear plate at a density of 2.5×10^3^ cells/well in 100 μl culture medium and allowed to attach for 24 h at 37°C. After incubation cells were treated with hydrogen peroxide at concentrations ranging from 0-500 μM (MRC-5) or 0-100 μM (H8F2p25LM). Working solution of hydrogen peroxide was prepared in culture medium suitable for appropriate fibroblast line. After 24 h exposure to hydrogen peroxide medium was removed, replaced with 100 μl of 2% Neutral Red solution and incubated for 1 h at 37°C. Then the cells were washed with PBS, fixed with 100 μl/well 50% ethanol, 49% H_2_O and 1% glacial acetic acid, and shaken for 20 min (700 rpm) at room temperature. Absorbance was measured at 540 nm against 620 nm.

### Antioxidant cytotoxicity

The cells were seeded in a transparent 96-well plate at a density of 5×10^3^ cells/well in 100 μl culture medium and allowed to attach for 24 h at 37°C. After incubation the cells were treated with different antioxidants (TEMPO, 4-amino TEMPO, 4-hydroxy TEMPO, melatonin, ethoxyquin, *p*-coumaric acid, ferulic acid, gallic acid, aminoguanidine hydrochloride, oleuropein, resveratrol, curcumin, caffeic acid) at 1, 10 and 100 μM concentrations. Working solutions of antioxidants were prepared in culture medium.

After 24 h exposure to antioxidants medium was removed and replaced with 100 μl of 1% Neutral Red solution and incubated for 1 h at 37°C. Than the cells were washed with PBS, fixed with 100 μl/well 50% ethanol, 49% H_2_O and 1% glacial acetic acid and shaken for 20 min (700 rpm) at room temperature. Absorbance was measured at 540 nm against 620 nm.

### Stress-induced premature senescence (SIPS)

SIPS studies were performed on cells grown at a density of 5 x 10^3^ cell/well (both MRC-5 and H8F2p25LM), 24 h post-seeding. After incubation cells were treated with H_2_O_2_ at 50% inhibitory concentration (IC_50_) for 24 h at 37°C to induce senescence. Working solutions of H_2_O_2_ were prepared in culture medium (DMEM or DMEM+GlutaMax). After 24 h the cells were posttreated with studied antioxidants at various concentrations depending of substances (ranging from 0 to 100 μM) for 24, 48 or 96 h. Cells treated with H_2_O_2,_ but not posttreated with antioxidants were used as a control. Subsequently, fibroblast viability was tested using NR assay, as a described above.

### Catalase activity

Fibroblast cells (MRC-5, H8F2p25LM) were seeded in transparent 6-well plate and allowed to attach for 24 h at 37 °C. After incubation the cells were trypsinized and their number was counted. Then the cells were transferred to a 15 ml falcon, centrifuged at 900 rpm for 5 minutes, washed with PBS and centrifuged once again. After these steps, supernatant was removed and 50 mM phosphate buffer (pH 7) with 0.1% Triton X-100 was added. The samples were centrifuged at 14000 g for 25 minutes at 0°C and supernatant was collected and used for further determinations. 0.036% solution of H_2_O_2_ in 50 mM phosphate buffer was introduced into a quartz cuvette (1 ml). Then the supernatant from cell lysis was added and absorbance was measured at 240 nm for 5 minutes (kinetic measurement).

### Content of reduced glutathione

To investigate the content of reduced glutathione in the fibroblasts, the method with *ortho*-phtal-dialdehyde was used. The cells were seeded in a transparent 96-well plate at an amount 7.5×10^3^/well and allowed to attach for 24 h at 37°C. After that, fibroblasts were treated with H_2_O_2_ at 600 μM concentration for MRC-5 and 35 μM for H8F2p25LM. After 24 h incubation, the medium was removed and replaced with antioxidant solutions: 1 μM curcumin, 2 μM 4-amino TEMPO, 10 μM caffeic acid and 50 μM p-coumaric acid. These concentrations were based on the effects of antioxidants on the survival of H_2_O_2_-treated cells. Tests were performed after 24 h, 48 hand 96 h incubation with studied substances. After incubation antioxidant solutions were removed, the cells were washed with PBS (150 μl per well) and then 60 μl/well of cold lysis buffer (RQB buffer: 20 mM HCl, 5% TCA, 5 mM DTPA, 10 mM L-ascorbic acid) were added. The plate was shaken for 5 minutes and centrifuged at 4000 rpm for 5 minutes. Next the cell lysate was transferred into two 96-well black bottom plates (‘+ NEM’ and ‘- NEM’) in an amount of 25 μl/well. Within the plate ‘+ NEM’, 4 μl/well freshly prepared 7.5 mM NEM in cold RQB buffer were added. Then 40 μl/well 1 M phosphate buffer (pH 7.0) were added into both plates and the plates were shaken for 5 min at 700 rpm. Subsequently, 160 μl/well of cold 0.1 M phosphate buffer (pH 6.8) and 25 μl/well of freshly prepared 0.5% OPA in methanol were added into both plates. Both plates were incubated for 30 minutes at room temperature under constant stirring. Fluorescence was measured at 355/430 nm [31]. The content of reduced glutathione was determined by subtracting the fluorescence of the ‘- NEM’ plate from the fluorescence ‘+ NEM’ plate and calculated with respect to the protein content. Protein content in cell lysates was determined according to Lowry et al. [32]. The results were expressed as a percentage of untreated control.

### Fluorometric measurement of reactive oxygen species (ROS) with H_2_DCF-DA

The level of ROS in H_2_O_2_-treated fibroblasts posttreated with antioxidants was assayed with 2′,7′-dichlorofluorescein (H_2_DCF-DA). The cells were seeded in 96-well flat clear-bottom black plate at an amount of 7.5×10^3^/well and allowed to attach for 24 h in 37°C. After that, fibroblasts were treated with H_2_O_2_ at 600 μM concentration for MRC-5 and 35 μM for H8F2p25LM. After 24 h incubation, the medium was removed and replaced with antioxidants solutions: 1 μM curcumin, 2 μM 4-amino-TEMPO, 10 μM caffeic acid or 50 μM *p*-coumaric acid. Tests were performed after 24 h, 48 h and 96 h incubation with studied substances. After incubation with antioxidants the medium was removed and replaced by 10 μM 2′,7′-dichlorofluorescein (H_2_DCF-DA) (100 μl/well). Stock H_2_DCF-DA solution was prepared in DMSO, and working solution was prepared in phosphate buffer. Fluorescence was measured at 490/529 nm for 2 h at 37°C (fluorescence measurements every minute).

### Fluorometric measurement of mitochondrial superoxide radical level

To estimate the level of superoxide in H_2_O_2_ treated cells posttreated with antioxidants, Cell meter TM Fluorimetric Mitochondrial Superoxide Activity Assay kit from AAT Bioquest (cat. no. 22971) was used. Briefly, cell were seeded in 96-well flat clear-bottom black plates at a density of 7.5×10^3^/well and allowed to attach for 24 h at 37°C. After incubation cells were treated with hydrogen peroxide (24 h) and posttreated with selected antioxidants (24, 48, 96 h) as described earlier. Subsequently the medium with antioxidants was removed, and 100 μl/well of MitoROSTM 580 working solution was added into the wells. Cells were incubated at 37°C for 45 minutes. Then, incubation fluorescence was measured at 540/590 nm for 4 h at 37°C (fluorescence measurements every minute).

### Mitochondrial membrane potential (Δψ_m_)

To estimated changes in the mitochondrial membrane potential after the use of selected antioxidants, JC-1 (5,5′,6,6′-tetrachloro-1,1′,3,3′-tetraethylbenzimida-zolylcarbocyanine iodide) with a Mitochondrial Membrane Potential Assay kit from Abnova was used. JC-1 is a lipophilic, cationic dye that can selectively enter the mitochondria and reversibly change color from green to red as the membrane potential increases. In cells with high *Δψ_m_*, JC-1 spontaneously forms complexes known as J-aggregates with intense red fluorescence. However, in injured cells with low *Δψ_m_*, JC-1 remains in the monomeric form and shows only green fluorescence.

Briefly, fibroblasts were seeded in a 96-well flat clear-bottom black plate at a density of 7.5×10^3^/well and allowed to attach at 37°C for 24 h. After incubation the cells were treated with H_2_O_2_ (24 h) and posttreated with selected antioxidants as described earlier. Then 10 μl of JC-1 staining solution were added into plate wells and the plate was incubated at 37°C for 30 min. After this time the cells were centrifuged at 4000 rpm for 5 min and the supernatant was gently removed. The plate was washed twice using the buffer included in the kit and centrifuged at 4000 rpm for 5 min. The supernatant was replaced by the buffer (100 μl/well) and fluorescence was measured at 535/595 nm (J-aggregates) and 485/535 nm (J-monomers). Data are shown as a ratio of fluorescence of J-aggregates to that of J-monomers.

### Mitochondrial mass

MRC-5 and H8F2p25LM cells were seeded at an amount of 1×10^5^ cells/well into a 6-well plate and allowed to attach at 37°C. After incubation the cells were treated with H_2_O_2_ (24 h) and posttreated with selected antioxidants (curcumin, 4-amino-TEMPO, caffeic acid, *p*-coumaric acid) for 24, 48, 96 h. Subsequently the cells were trypsinized, counted, transferred to Eppendorf tubes and centrifuged for 5 minutes at 2000 rpm, then washed with 1 ml of PBS and centrifuged again. Subsequently, 1 ml of 10 μM N-nonyl acridine orange (NAO) in PBS was added and the cells were incubated at 37°C for 10 min. After incubation with NAO cells were centrifuged and washed with PBS (1 ml) and then the cell pellet was resuspended in 300 μl of PBS. Each sample was transferred into a 96-well black plate (100 μl/well, 3 repetitions). Fluorescence was measured at 435/535 nm. The results were calculated respectively to cell number.

### Staining cells for senescence-associated β-galactosidase

2×10^5^ MRC-5 cells and 1.4×10^5^ H8F2p25LM cells were seeded into wells of 6-well plates and allowed to grow for 24 hours. Then the medium was exchanged for a fresh one containing H_2_O_2_ in a final concentration of 600 μM or 35 μM, respectively (except for control wells) and the cells were incubated for further 24 hours. The medium was again replaced by a fresh one containing 1 μM curcumin, 2 μM 4-amino-TEMPO, 10 μM caffeic acid or 50 μM coumaric acid. Cells were harvested after 24, 48 or 96 hours since the last medium exchange, centrifuged (100×g for 10 minutes, room temperature), resuspended in 200 μL of 1 μM 4-methylumbelliferyl β-D-galactopyranoside dissolved in fresh medium, incubated for 1 hour at 37°C and then analysed by flow cytometry (excitation: 355 nm, emission: 425-475 nm). Median fluorescence of control samples at 24-hour posttreatment was assumed as 100%.

### Statistical analysis

Kruskal-Wallis test or t-Student test was performed to estimate differences between H_2_O_2_ treated control cells and antioxidant posttreated cells to assess their properties in each individual assay; P≤ 0.05 was considered as statistically significant in both cases. Also differences between H_2_O_2_-treated and non-treated controls were assessed by an appropriate test (one of two described above). Statistical analysis of the data was performed using STATISTICA software package (version 13.1, StatSoft Inc. 2016, Tulsa, OK, USA).
